# Multisystem inflammatory syndrome in children associated with COVID-19: a case report and literature review

**DOI:** 10.11604/pamj.2025.51.96.40535

**Published:** 2025-08-19

**Authors:** Mohamedraza Akbar Ebrahim, Shadrack Elia Makundi, Noureen Ndelimbi Mushi, Pendo Mussa Ibrahim, Rukhsar Shabir Osman, Zainab Fidaali, Deogratias Arnold Nkya

**Affiliations:** 1 The Aga Khan University, Dar es Salaam, Tanzania,; 2 The Aga Khan Hospital, Dar es Salaam, Tanzania,; 3 Muhimbili University of Health and Allied Sciences Dar es Salaam, Tanzania

**Keywords:** Severe acute respiratory syndrome coronavirus 2, multisystem inflammatory syndrome, COVID-19, case report

## Abstract

Given the rarity of multisystem inflammatory syndrome related to COVID-19 (MIS-C). This case report highlights the high index of suspicion to diagnose MIS-C in a child who presented with symptoms suggestive of acute gastroenteritis. While admitted in the hospital, she deteriorated with worsening of symptoms, clinical decline, deranged laboratory markers, and significant radiological findings, even though on antibiotics. She had acute myocarditis with myocardial dysfunction on echocardiography and raised cardiac enzymes. Intravenous human immunoglobulin (IVIG) was given, but there was additional need for inotrope (norepinephrine) and methylprednisolone albeit the preliminary echocardiogram was normal. Early recognition of MIS-C with rapid escalation of care post IVIG administration is needed to prevent morbidity and mortality.

## Introduction

The Coronavirus disease (COVID-19) caused by severe acute respiratory syndrome coronavirus 2 (SARS-CoV-2) according to the World Health Organization (WHO) has affected more than 700 million people and caused more than 6 million deaths by March 31^st^, 2023. The prevalence of COVID-19 in children ranges between 10 and 30% and most of them are asymptomatic or mildly symptomatic. Less than 1% develop a rare complication called Multisystem Inflammatory Syndrome associated with COVID-19 (MIS-C) as defined by the WHO [1]. The disease presents with life-threatening cardiovascular shock, high-grade fever, and hyperinflammation with clinical manifestations of atypical Kawasaki disease, Kawasaki disease shock syndrome, and bacterial toxic shock syndrome [2,3]. In April 2020, the first series of eight children with MIS-C was described in the United Kingdom [4].

Several theories have been postulated to describe the pathogenesis of MIS-C, and among them are the immune dysregulation theory and molecular mimicry theory. In immune dysregulation theory, early infection activates macrophages and T-helper cell stimulation, leading to a burst of cytokine release from cells (cytokine storm). B-cells and plasma cells are also activated and produce antibodies, which causes a hyper-immune state. This hypothesis is supported by the fact that the majority of children with MIS-C have COVID-19 positive serology and negative polymerase chain reaction (PCR) test results. The other hypothesized mechanism is the molecular mimicry theory, in which antibodies against the COVID-19 antigenic parts of the viral structure cross-react with the patient´s own body cells. This mechanism leads to a delayed T-cell autoantibody response occurring 4 - 6 weeks after the COVID-19 infection caused by circulating autoantibodies and immune complexes. This mechanism is supported by the presence of autoantibodies against the immune cells, endothelial cells, and gastrointestinal cells, but also the fact that there is clinical evidence of multisystem involvement in MIS-C [5]. We are presenting a case of a 9-year-old girl with MIS-C that was successfully treated in our facility.

## Patient and observation

**Patient information:** a 9-year-old African girl presented with a four-day history of high-grade fever, headache, redness of the eyes, nausea, vomiting, and abdominal pain. The vomitus consisted of recently eaten food material and occasionally bilious matter; she had 3-4 episodes in a day. No cough, chest pain, chest tightness, or difficulty in breathing. She was taken to a nearby hospital, where laboratory workup was done and started treatment (tablets amoxicillin and clavulanic acid 625mg 12 hourly and ondansetron tablets 8mg every 8 hours), but had no symptom relief. A prior history of flu-like symptoms with nasal congestion, cough, body weakness, and cracking of the lips.

**Clinical findings:** assessment at the emergency department revealed a sick-looking young girl with non-purulent conjunctivitis, jaundice, and dry lips with normal peripheral perfusion. She had stable vital signs except for tachycardia. She also had generalized abdominal tenderness without distension. The initial laboratory assessment showed elevated C-Reactive protein (CRP = 200mg/dL) and thrombocytopenia (84 x10^9^/L). Other white blood cell counts were normal.

### Timeline of current episode

**Figure 1:**
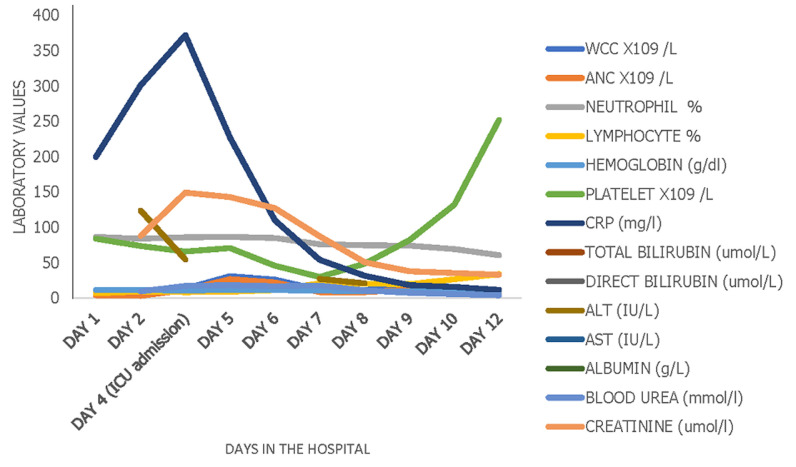
trend of laboratory markers from the day of hospital admission to the day of discharge

**Figure 2:**
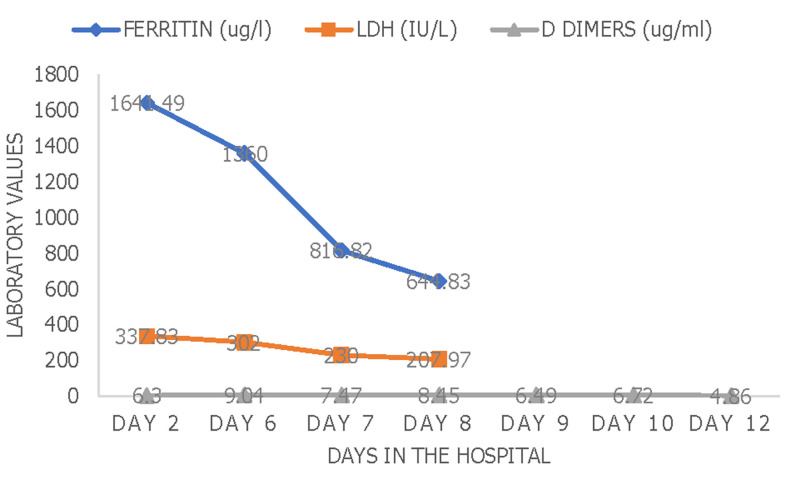
dynamic changes of inflammatory markers of the patient during the course of hospital stay

**Day 1 to 2:** there were persistent fevers, headache, abdominal pain, conjunctivitis, diarrhea, and cracking of the lips. Abdominal ultrasound showed hepatosplenomegaly with reactive cholecystitis. Laboratory marker CRP-301 mg/dL and the COVID-19 antibody IgG test were positive (SGTi-flex COVID-19 IgM/IgG). Intravenous antibiotics (ceftriaxone 75 mg/kg/day) were continued. A pediatric cardiologist review and an echocardiogram showed a left ventricular ejection fraction was 70% with fractional shortening of 36%. Based on the history, clinical findings, laboratory (Figure 1), inflammatory markers (Figure 2), and radiological report. This child was given intravenous immunoglobulins (IVIG) 2g/kg over 12 hours and aspirin 75mg once daily.

**Day 3:** she developed cold extremities, hypotension (BP = 60/30 mmHg), tachycardia (HR = 154 bpm), tachypnea (RR = 45 bpm), bilateral posterior basal crepitations, hepatomegaly, and depressed level of consciousness with the Glasgow Coma Scale (GCS) of 8/15. The laboratory markers CRP increased to 372 mg/dL, and thrombocytopenia to 66 x10^9^/L. Chest X-ray showed pulmonary edema and cardiomegaly. The change in her clinical deterioration was suggestive of cardiogenic shock. Norepinephrine peripheral dose infusion was initiated at 0.05 mcg/kg/min, and she was transferred to the pediatric intensive care unit (PICU) for close monitoring. The septic workup was done, and antibiotics were escalated to meropenem 20mg/kg/day every 8 hours.

**Day 4:** there was an apparent increase in blood urea nitrogen and creatinine. Repeat echocardiogram findings depressed left ventricular function, thin myocardial walls, mild mitral and tricuspid regurgitation, with an ejection fraction of 47% and fractional shortening 23%. Considering the new findings, a diagnosis of acute myocarditis was established. She was started on intravenous furosemide (2mg/kg/day), oral spironolactone (2mg/kg/day), and intravenous methylprednisolone (4mg/kg/day) for MIS-C.

**Figure 3:**
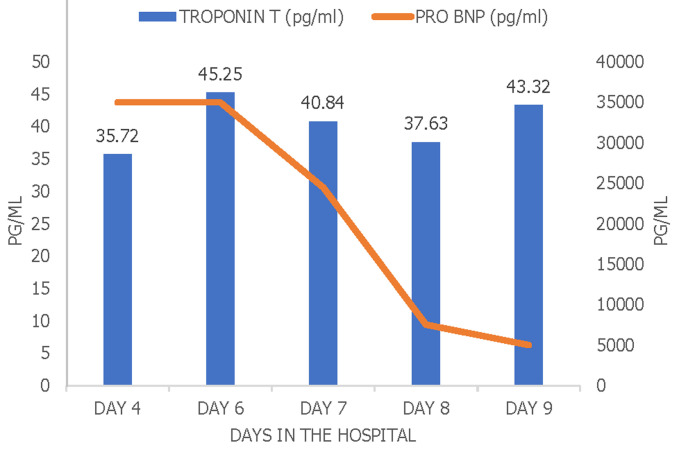
levels of cardiac biomarkers of the patient in the course of hospital stay

**Day 6:** marked improvement of abdominal pain and conjunctivitis. Troponin T levels reached a maximum of 45.2 pg/mL (Figure 3). The child was weaned off inotropes, and a repeat echocardiogram was done, which showed a mildly dilated left atrium and ventricle. Improving left ventricular function with an ejection fraction of 55-60% and a fractional shortening of 32%. She was seen by a physiotherapist for mobilization and rehabilitation. She was started on fluconazole (6mg/kg/day) systemic fungal prophylactic dose while in the pediatric ICU.

**Day 7:** received platelet transfusion due to thrombocytopenia (30 x10^9^/L).

**Day 8:** transferred back to the pediatric ward and continued with medications.

**Day 11:** repeat echocardiogram showed resolved myocardial function with ejection fraction of 64% and fraction shortening of 35%.

**Day 12:** discharged home on oral prednisolone, aspirin, and diuretics (furosemide and spironolactone).

**Diagnostic assessment:** the worsening clinical condition, despite appropriate use of antibiotics, raising CRP, worsening thrombocytopenia, positive COVID-19 antibody test, and radiological investigations revealed multisystem involvement, raising suspicion of MIS-C.

**Diagnosis:** she had acute myocarditis, which was evidenced by acute myocardial dysfunction on echocardiography and elevated cardiac enzymes.

**Therapeutic interventions:** this child was treated with intravenous ceftriaxone 75mg/kg for 72 hours and afterward intravenous meropenem 20mg/kg every 8 hours for 7 days (sepsis), analgesics for pain, and norepinephrine infusion for cardiogenic shock. She was transfused with platelet concentrates for thrombocytopenia. High-dose aspirin was begun at the dose of 50mg/kg for five days, then followed by low-dose aspirin at 5mg/kg once a day for eight weeks. She was also given pantoprazole to prevent gastrointestinal effects of aspirin.

**Follow-up and outcome of interventions:** monitored outpatient by pediatric cardiologist, and a subsequent echocardiogram was done, which revealed normal myocardial function. Gradually weaned off diuretics, oral steroids, and aspirin.

**Patient’s parent perspective:** we were very much impressed by the high degree of professionalism in handling of case, from diagnosis to treatment. On the course of treatment, it was very encouraging to see tests done daily to monitor the identified problems and provide feedback to us as parents. The fact that we were able to see her improvement daily, both physically and from the test results, was proof of the right treatment she was receiving. Had it not been for the timely identification of the disease complication and the decision on the right treatment route, we would have lost our beloved daughter. Our special thanks to the entire crew for saving our daughter´s life, and we thank God for leading you all in what you did on this case.

**Informed consent:** written consent was obtained from the parents.

## Discussion

Multisystem inflammatory syndrome post COVID-19 (MIS-C) is a worldwide phenomenon affecting both children and adults (MIS-A). This syndrome is also known as pediatric multisystem inflammatory syndrome. The clinical features of MIS-C mimic those of Kawasaki disease (KD) and were first reported in children with COVID-19 in the United Kingdom (UK) in April 2019. Cases of MIS-C have been reported worldwide with varying clinical presentations, treatment modalities, and outcomes. Few cases have been reported from the African continent.

Different studies from the United States and South Africa report black children to be at a significantly higher risk of testing positive for SARS-CoV-2, and having higher rates of severe COVID-19 infection, including MIS-C. A South African study showed 80% of children with MIS-C were black, whereas in the United States, 56% positive evaluated cases for COVID-19 in one study, and 35% of children with MIS-C in another study, were black children [6,7].

The index case was symptomless for COVID-19, had no known exposure to symptomatic or reverse transcription polymerase chain reaction (RT-PCR) positive individuals, however, her typical presentation with fever, myalgia, abdominal symptoms, mucocutaneus involvement, and elevated inflammatory markers raised suspicion for multisystem inflammatory syndrome. She tested negative for RT- PCR for SARS-CoV-2 and other infections (Table 1). However, antibody IgG for SARS-CoV-2 was positive, and presented several weeks after peak transmission of SARS-CoV-2 in our region. This is coherent with multiple reports and reversible outcomes that suggest MIS-C is a post-infectious syndrome, related to host immune response and hyperinflammation.

**Table 1:** results of screened infections among our patients demonstrating the absence of other infectious diseases

Malaria	Negative
Dengue	Negative
HIV	Negative
Hepatitis B surface antigen	Negative
Hepatitis C antibodies	Negative
Blood culture	Negative
Urine culture	Negative
Tropical fever panel PCR	Negative
Fever with rash, PCR panel	Negative
COVID-19 PCR test	Negative
COVID-19 antibodies	Positive

PCR: polymerase chain reaction

Whilst most of our clinical features are consistent with reported case series and literature, an interesting observation is that this child´s initial transthoracic echocardiogram prior to administration of IVIG was normal, however, her cardiac function rapidly deteriorated. Subsequently, the patient required inotropic support and transfer to the PICU. The serial echocardiogram findings and cardiac biomarkers are consistent with reported verdicts in multiple case series [4,8,9,10].

## Conclusion

To prevent the consequences of the multisystem inflammatory Syndrome associated with COVID-19 (MIS-C), an uncommon but fatal disease, early diagnosis and prompt management are essential.
